# Evaluation of the effects of four media on human intestinal microbiota culture in vitro

**DOI:** 10.1186/s13568-019-0790-9

**Published:** 2019-05-23

**Authors:** Fu Yousi, Chen Kainan, Zhang Junnan, Xiao Chuanxing, Fan Lina, Zhang Bangzhou, Ren Jianlin, Fang Baishan

**Affiliations:** 1 Department of Chemical and Biochemical Engineering, College of Chemistry and Chemical Engineering, Xiamen University, No. 422, Siming South Road, Xiamen, 361005 Fujian China; 2 Zhongshan Hospital Department of Gastroenterology, Xiamen University, No. 201-209, Hubinnan Road, Xiamen, 361005 Fujian China

**Keywords:** Intestinal microbiota, Short chain fatty acid, In vitro culture, 16S rRNA

## Abstract

The human intestinal microbiota has an important role in the maintenance of human health and disease pathogenesis. The aim of this research was to investigate the impact of four media on human intestinal microbiota metabolite and composition changes, we performed in vitro batch culture using intestinal microbiota samples from three fecal microbiota transplantation (FMT) donors. After 48 h culture, gut microbiota medium (GMM) had the highest production of acetic acid (73.00 ± 7.56 mM) and propionic acid (16.79 ± 1.59 mM), bacterial growth media (BGM) had the highest production of butyric acid (13.39 ± 0.56 mM). In addition, brain heart infusion (BHI) promoted (p < 0.05) the growth of *Bacteroidetes*, especially *Bacteroides* after 48 h, GMM resulted in a significant increase (p < 0.05) in *Actinobacteria* and increased the beneficial genus *Bifidobacterium*, fastidious anaerobe broth (FAB) increased *Firmicutes* population, and BGM promoted the growth of *Escherichia*–*Shigella* and *Akkermansia*. The results suggest that four media had different effects on the human intestinal microbiota metabolism and composition in vitro. These results may facilitate the culture of bacteria from the human intestinal microbiota.

## Introduction

The human gastrointestinal tract harbors at least 10^14^ bacterial cells of 400–1000 bacterial species to form the intestinal microbiota that has an important role in the maintenance of human health (Eckburg et al. 2005; Tremaroli and Bäckhed 2012). Interactions of the human microbiota with the host are usually mediated by bacterial metabolic products such as vitamins, amino acids and short chain fatty acid (SCFA) (Tramontano et al. 2018). Changes in the equilibrium of the gut microbial ecosystem have been associated with a range of diseases, including obesity, type II diabetes and inflammatory bowel disease (Makki et al. 2018; Soto et al. 2018). Fecal microbiota transplantation (FMT) is a new method to treat a variety of dysbiosis-associated gut diseases, such as *Clostridium difficile* infection (CDI) (Surawicz et al. 2013) and chronic hepatitis B (Ren et al. 2017). FMT involves transfer of intestinal microbiota from healthy donors to patients to correct intestinal microbiota dysbiosis (Staley et al. 2018). The intestinal microbiota from FMT donors can be used to represent the healthy human intestinal microbiota (Gupta and Khanna 2017).

Culturomics is a culturing approach that uses multiple culture conditions, 16S rDNA sequencing and MALDI–TOF mass spectrometry for the identification of bacterial species (Lagier et al. 2018). The first step of culturomics is to diversify the intestinal microbiota sample into different culture media, promoting the growth of fastidious bacteria from the human gut; the next step is to optimize culture conditions to promote the growth of fastidious microorganisms at lower concentrations (Lagier et al. 2018). Anaerobes are dominant members of the human intestinal microbiota (Pham and Mohajeri 2018). In addition, culturing obligate anaerobes requires oxygen-free environment and complex media with many supplements (macroelements and growth factors) (Lau et al. 2016). In vitro batch culture is a semi-representative model and provides a time- and cost-effective way to culture human intestinal microbiota under simulated physiological conditions (anaerobiosis, culture medium, 37 °C) (Williams et al. 2015). The advantages of in vitro batch culture are fast, cheap, easy to operate and reproducible (Pham and Mohajeri 2018), and this approach can serve as high-throughput initial investigations of the culturomics by providing important clues to guide further studies.

Therefore, the objective of the present study was to investigate the effects of four culture media on human intestinal microbiota from FMT donors using an in vitro batch culture model. We envision that these results can improve our knowledge of human microbial ecosystem and facilitate human intestinal microbiota culture in vitro.

## Materials and methods

### Materials

High purity SCFA standards for gas chromatography (GC) analysis, including acetic acid, propionic acid and butyric acid, were purchased from Sigma Aldrich Chemical Co. (St Louis, MO, USA). l-Cysteine hydrochloride monohydrate, bile salts, tryptone, yeast extract, glucose and other chemicals were obtained from Sangon Biotech (Shanghai, China). Media used in this study included: brain heart infusion (BHI) media (Solarbio, Beijing, China), gut microbiota medium (GMM) without agar (Goodman et al. 2011), fastidious anaerobe broth (FAB, Solarbio, Beijing, China), bacterial growth media (BGM) (Mcdonald et al. 2013). All media were prepared following the manufacturer’s instructions.

### Preparation of human intestinal microbiota

Three human intestinal microbiota samples were collected at Zhongshan Hospital of Xiamen University from healthy FMT donors who had not received antibiotics within 3 months of sampling, and had no history of digestive disease (Ren et al. 2017). Intestinal microbiota samples from three donors were pooled after collection. The study was approved by Xiamen University and Zhongshan Hospital of Xiamen University, China. All FMT donors provided written informed consent. In vitro culture was performed in compliance with the relevant laws and institutional guidelines.

### In vitro culture

In vitro culture was performed in 150 mL volumes of sterilized culture medium in 250 mL containers. Sterilized culture medium was added to the vessels, which were then maintained at 37 °C with magnetic stirring at 150 rpm. Prior to culture, the vessels were sparged with N_2_ gas to obtain an anaerobic environment. The following day, 15 mL of fecal inoculum were inoculated into each container. Batch culture was carried out anaerobically for 48 h at 37 °C, with samples collected at seven time points (0, 4, 8, 12, 24, 36 and 48 h) over the course of the experimental period. Samples were stored at − 80 °C until further analysis.

### Determination of SCFA content

For SCFA analysis, samples were filtered through a 0.22 μm membrane prior to analysis. The SCFA content was analyzed using an Agilent 7890A gas chromatograph with flame ionization detector. A gas chromatography column (AT-FFAP, 1116–342, 30 m × 0.32 mm i.d. ATEO, Lanzhou, China) coated with a 0.25 μm thickness film was used for the analysis. Chromatographic analysis was performed according to the manufacturer’s instructions. The initial oven temperature was 80 °C for 5 min, which was raised to 250 °C at 5 °C per min. The temperatures of the injection port and the flame ionization detector were 250 °C and 260 °C, respectively. The flow rate of the carrier gas, nitrogen, was set at 19.0 mL/min, with a split ratio of 1:10. The flow rates of hydrogen and air were 30 mL/min and 300 mL/min, respectively. The injection volume was 1 μL. Each sample was examined three times independently.

### DNA extraction and 16S rRNA gene sequencing

Samples (1.5 mL each) collected from culture vessels were centrifuged at 13,000×*g* for 10 min, the supernatant discarded, and the pellets were resuspended in 750 μL of bead solution. DNA extraction was then carried out using a Power Fecal DNA Isolation Kit (12830-50; MOBIO Laboratories, Carlsbad, CA, USA) according to manufacturer’s instructions. Primers F341 (5′-CCTAYGGGRBGCASCAG-3′) and R806 (5′-GGACTACNNGGGTATCTAAT-3′) were then used to amplify the V3–V4 domain of the bacterial 16S rRNA gene. PCR reactions contained 100–300 ng of template, 10× buffer (Toyobo, Osaka, Japan), 2 mM dNTPs, 25 mM MgSO_4_, 10 mM forward and 10 μM reverse primers, 1 μL of KOD enzyme, and sterile double-distilled H_2_O in a final volume of 50 μL. Reaction conditions consisted of an initial denaturation at 94 °C for 2 min, followed by 28–35 cycles of 98 °C for 10 s, 62–66 °C for 30 s, and 68 °C for 30 s, and a final extension of 68 °C for 5 min. The resulting amplicons were purified using AMPure XP beads (Beckman Coulter, Brea, CA, USA). Purified libraries were sequenced using the Illumina Hiseq 2500 platform (Illumina, San Diego, CA, USA). Raw Illumina read data for all samples were deposited in the NCBI Sequence Read Archive database under accession number PRJNA506872.

### Community structure analysis

Analysis of raw Illumina fastq files was performed using quantitative insights into microbial ecology 2 (Qiime 2, ver. 2018.2 https://qiime2.org/). Sequences were quality filtered, trimmed, de-noised, and merged using DADA 2 (Callahan et al. 2016). Chimeric sequences were then identified and removed, taxonomy was classified using the Silva 119 16S rRNA database (Quast et al. 2013). To analyze the alpha diversity, Shannon index and observed OTUs were calculated by QIIME 2. For the beta diversity analysis, constrained principal coordinates analysis (CPCoA) of Bray–Curtis distances constrained by group was computed using the capscale function implemented in the vegan R library (version 2.5-2). In order to mine deeper data of microbial diversity of the difference among the four groups, significance test was conducted with linear discriminant analysis effect size (LEfSe, http://huttenhower.sph.harvard.edu/galaxy). The network was generated using the CoNet plugin (version 1.1.1) for Cytoscape (version 3.6.1) on the basis of the nonparametric Spearman correlation coefficients, with a minimal cutoff threshold rho of 0.6 (p < 0.05, Benjamini–Hochberg corrected) (Yan et al. 2018).

### Statistical analysis

Data were expressed as mean ± standard deviation (SD). All of the four groups were conducted with twelve same intestinal microbiota samples. Statistical comparisons of SCFA production and alpha diversity data were evaluated by one-way analysis of variance, and compared using the Tukey test at 5% confidence level using SPSS software version 22.0 (SPSS, Chicago, IL, USA). Values of p < 0.05 were considered statistically significant. Venn diagrams and constrained principal-coordinate analysis (CPCoA) were created in the R software (version 3.5.0).

## Results

### Comparison of media composition

**Fig. 1:**
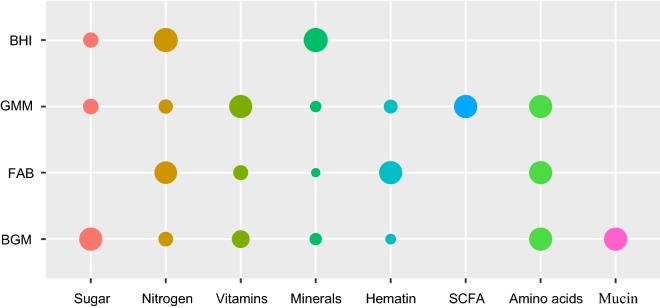
Comparison of nutrient group representation across four media. Circle size correlates linearly with the quantity of the respective nutrient group

In this study, the human intestinal microbiota from FMT donors was cultured in four types of media including BHI, GMM, FAB and BGM. All media were composed of commercially available components (Fig. 1). BHI media consisted of sugar, nitrogen and the highest concentration of minerals in comparison with other three media. GMM media contained SCFA and the highest concentration of vitamins among four media. FAB media did not contain sugar components and BGM media contained mucin and the highest concentration of sugar among four media.

### SCFA production and pH shift

**Fig. 2:**
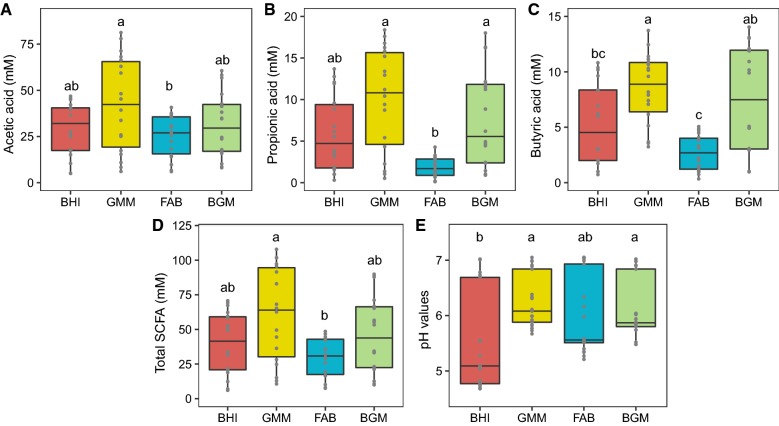
SCFA production from 4 to 48 h and pH values from 0 to 48 h of BHI, GMM, FAB and BGM groups. **A** Acetic acid, **B** propionic acid, **C** butyric acid, **D** total SCFA, **E** pH values. Data were analyzed using ANOVA with Tukey HSD (p < 0.05)

Anaerobic cultures were conducted using the four types of media in the presence of human intestinal microbiota at initial pH values of 7.0. The production (minus the concentration of SCFA at 0 h) of SCFA, including acetic, propionic and butyric acid is presented in Fig. 2. Acetic acid production from 4 to 48 h was similar for the BHI, FAB and BGM groups. The GMM group produced 73.00 ± 7.56 mM acetic acid after 48 h, which was significantly (p < 0.05) higher than that in the FAB group. Similarly, the GMM group produced the highest level of propionic acid (16.79 ± 1.59 mM), which was significantly (p < 0.05) higher than that in the FAB group. The highest production of butyric acid (13.39 ± 0.56 mM) was obtained in the BGM group, and significantly (p < 0.05) more than the FAB group. After 48 h, the production of total SCFA was significantly (p < 0.05) higher in the GMM group compared to that in the FAB groups. The pH shift as a reflection of culture was evaluated (Fig. 2E). In the BHI group, the pH value decreased to 4.80 ± 0.02 after 48 h, which was significantly (p < 0.05) lower than that in the GMM and BGM groups. Furthermore, there were no significant (p > 0.05) differences in pH values among the GMM, FAB and BGM groups from 0 h to 48 h.

### Composition of microbial community

**Fig. 3:**
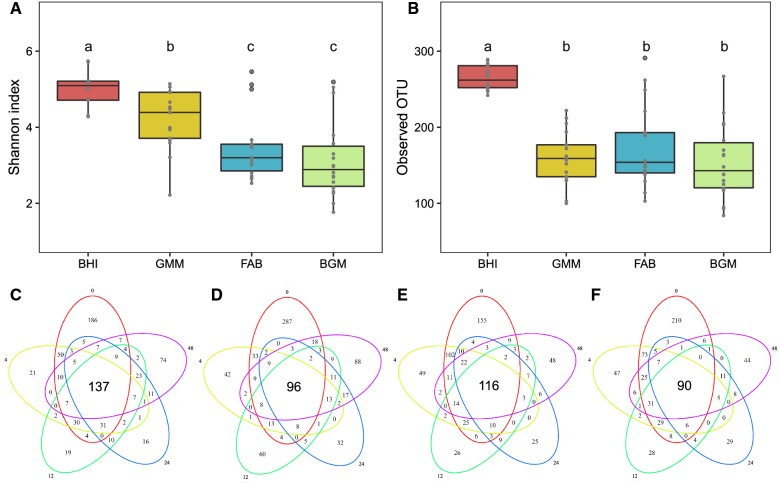
Community diversity as measured by the Shannon index (**A**) and Observed OTUs (**B**) of the BHI, GMM, FAB and BGM groups from 4 to 48 h culture. Venn diagrams illustrating the number of unique and shared OTUs among BHI (**C**), GMM (**D**), FAB (**E**) and BGM (**F**) groups at 0, 4, 12, 24, 48 h

**Fig. 4:**
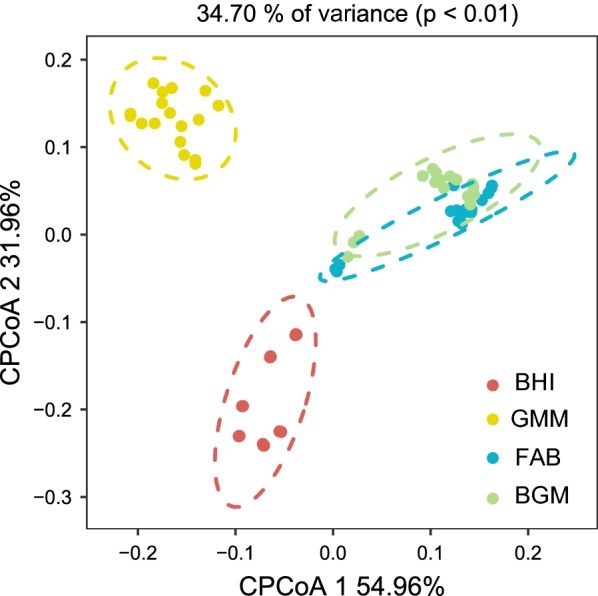
Constrained PCoA plot of Bray–Curtis distances of the BHI, GMM, FAB and BGM groups constrained by group (34.70% of variance explained, p < 0.01) from 4 to 48 h

The intestinal microbial community structures in four groups were further investigated by high-throughput sequencing. Two alpha diversity measures were calculated including Shannon index and observed OTUs (Fig. 3A, B). For this, the Shannon index of the BHI group was significantly (p < 0.05) higher than that of other three groups (Fig. 3A). Regarding the comparison of community richness, the BHI group had a significantly (p < 0.05) higher number of observed OTUs than other three groups (Fig. 3B). The Venn diagram showing overlap in the observed OTUs among the samples revealed that there were 137 shared OTUs in the BHI group (Fig. 3C), 96 in the GMM group (Fig. 3D), 116 in the FAB group (Fig. 3E) and 90 in the BGM group (Fig. 3F) from 0 to 48 h culture. A higher number of OTUs were retained in the BHI group compared to other three groups after 48 h culture. The analysis of constrained principal-component (CPCoA) indicated that there was a significant separation of the four groups, especially between the BHI and GMM groups from 4 to 48 h culture (Fig. 4). This analysis revealed that the bacteria communities differences between the BHI and GMM were larger than the differences between the FAB and BGM groups.

**Fig. 5:**
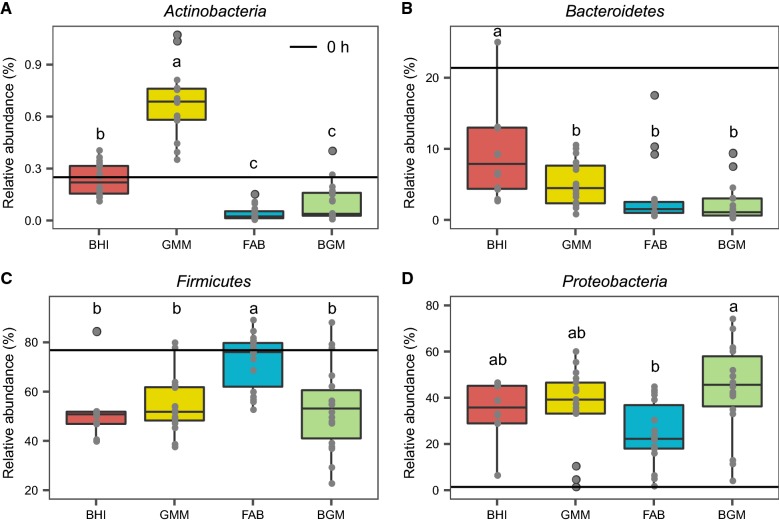
The relative abundance of the top 4 most abundant intestinal microbiota at the phylum-level in BHI, GMM, FAB and BGM groups from 0 to 48 h. **A** *Actinobacteria*, **B** *Bacteroidetes*, **C** *Firmicutes*, **D** *Proteobacteria*. Data were analyzed using ANOVA with Tukey HSD (p < 0.05)

The relative abundance of the top 4 most abundant intestinal microbiota at the phylum-level in the BHI, GMM, FAB and BGM groups is illustrated by histograms in Fig. 5. The relative abundance of the *Actinobacteria* phylum in the GMM group was significantly (p < 0.05) higher than BHI, FAB and BGM groups. The relative abundance of *Bacteroidetes* in the four groups from 4 to 48 h culture was decreased compared to 0 h (FMT donors intestinal microbiota samples), and *Bacteroidetes* of BHI group was significantly (p < 0.05) higher than that of other three groups. A significant increase of *Firmicutes* was observed in the FAB group when compared to other three groups from 4 to 48 h culture. Compared to 0 h, there was a notable increasing relative abundance of *Proteobacteria* in the four groups from 4 to 48 h, and *Proteobacteria* of BGM group was significantly (p < 0.05) higher than that of other groups.

**Fig. 6:**
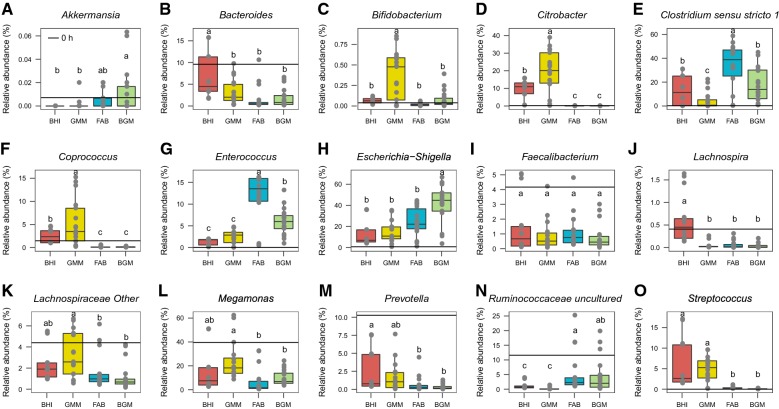
The relative abundance of 15 intestinal microbiota at the genus-level and in BHI, GMM, FAB and BGM groups from 0 to 48 h. **A** *Akkermansia*, **B** *Bacteroides*, **C** *Bifidobacterium*, **D** *Citrobacter*, **E** *Clostridium* sensu stricto 1, **F** *Coprococcus*, **G** *Enterococcus*, **H** *Escherichia*–*Shigella*, **I** *Faecalibacterium*, **J** *Lachnospira*, **K** *Lachnospiraceae Other*, **L** *Megamonas*, **M** *Prevotella*, **N** *Ruminococcaceae uncultured*, **O** *Streptococcus*. Data were analyzed using ANOVA with Tukey HSD (p < 0.05)

At the genus level (Fig. 6), a significant increase of *Clostridium*, *Enterococcus*, *Escherichia*–*Shigella* and *Streptococcus* and a significant decrease of *Faecalibacterium*, *Megamonas*, *Prevotella* and *Ruminococcaceae_un* were observed in the four groups from 4 to 48 h when compared to 0 h (p < 0.05). During the period from 4 to 48 h culture, the relative abundance of *Bacteroides* and *Lachnospira* in the BHI group was higher than that in other groups; the amounts of *Bifidobacterium* in GMM group ranging from 0.01 to 0.81% were significantly (p < 0.05) higher than other groups; the relative abundance of *Citrobacter* and *Coprococcus* in the GMM group was also higher than other groups (p < 0.05); there was a notable increasing relative abundance of *Clostridium* and *Enterococcus* in the FAB group compared to other three groups (p < 0.05); the BGM group indicated the highest relative abundance of *Akkermansia* and *Escherichia*–*Shigella* among the four groups. In addition, the relative abundance of *Faecalibacterium* in the four groups was similar from 4 to 48 h (p > 0.05).

**Fig. 7:**
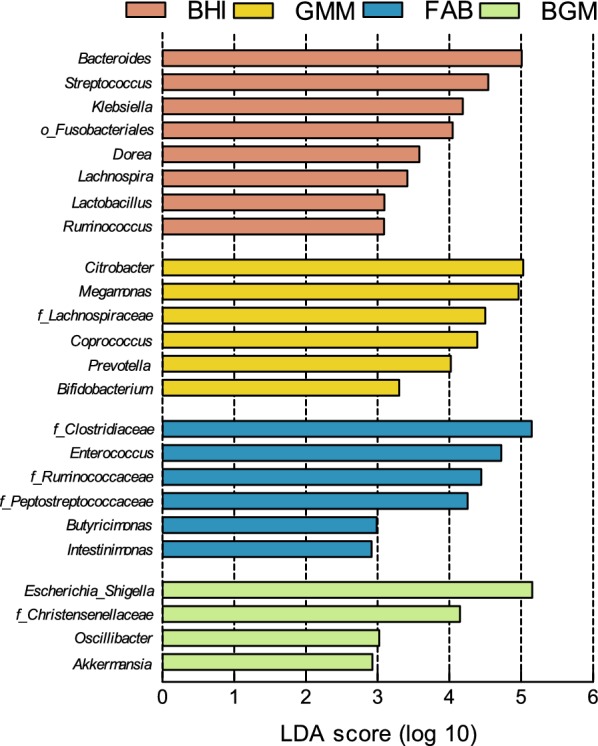
Bacterial taxa differentially enriched in BHI, GMM, FAB and BGM groups using linear discriminant analysis (LDA) effect size (LEfSe) algorithm from 4 to 48 h. LDA score is greater than 2

Additionally, bacterial taxa with significant difference among the BHI, GMM, FAB and BGM groups was demonstrated using LEfSe analysis (Fig. 7). LEfSe analysis indicated significant distinguishing bacteria with *Bacteroides*, *Streptococcus* and *Klebsiella* in the BHI group, with *Citrobacter*, *Prevotella*, *Coprococcus*, *Bifidobacterium* and *Megamonas* in the GMM group, with *Clostridiaceae*, *Enterococcus* and *Butyricimonas* in the FAB group, and with *Escherichia*–*Shigella*, *Christensenellaceae*, *Oscillibacter* and *Akkermansia* in the BGM group when compared with the other three groups.

**Fig. 8:**
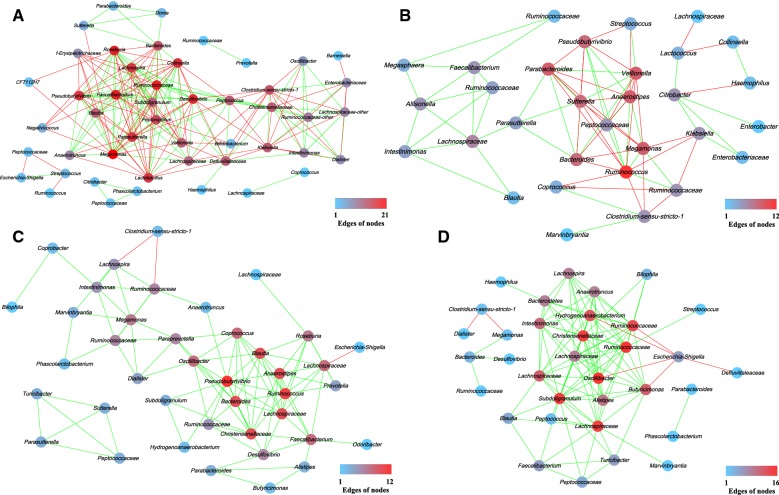
Network analysis of the BHI (**A**), GMM (**B**), FAB (**C**) and BGM (**D**) samples indicating interactions among dominant bacteria from 4 to 48 h. Nodes represent genus. The color of each node is proportional to the number of edges

To study the possible interactions among intestinal microbiota in the four groups, the relative abundance of genus higher than 0.1% were used to construct a network. As shown in Fig. 8, each node represents a bacterial genus and edges indicate significant co-occurrence (green) or mutual exclusion (red) interactions. Both significant co-occurrence and mutual exclusion (Spearman’s rho > 0.6) were observed in the four groups, confirming the presence of an interactive network of microorganisms. The network of the BHI group included 48 nodes and 203 edges. In this network, *Megamonas* had 21 edges, which were the key genus in the BHI group. The network of GMM group consisted of 31 nodes and 75 edges, and *Ruminococcus* were the nodes of the highest edges. In the FAB group network, 40 nodes and 103 edges were found, and *Pseudobutyrivibrio* (15 edges) were considered to be potential key genus. The network of BGM group included 34 nodes and 107 edges, *Ruminococcaceae_un* and *Oscillibacter* had 15 edges, which were the core genus in the BGM group.

## Discussion

The aim of this study was to investigate the impact of four media upon human intestinal microbiota metabolite and composition in anaerobic batch cultures inoculated with human intestinal microbiota samples from FMT donors. SCFA produce by intestinal microbiota leads to many beneficial health outcomes to the host. The higher SCFA concentration created a lower pH environment in gut which may inhibit pathogen colonization (Jin et al. 2018). Acetic acid is the main metabolite of most intestinal microbiota, and it is an important source of energy for tissues and the substrate of cholesterol synthesis (Wang et al. 2018). Propionic acid is also a very important SCFA in the intestinal tract, and it has been reported that propionic acid could impact glucose metabolism in intestinal epithelium (Zadehtahmasebi et al. 2016). After 48 h culture, the GMM group produced the highest concentration of acetic acid and propionic acid among four groups. This result suggested that GMM media may facilitate the production of acetic and propionic acid from intestinal microbiota. Among all SCFA, butyric acid is considered a source for the intestinal epithelial cells and is implicated in reducing the incidence of colon inflammatory processes (Thomson et al. 2018). The media that promoted by far the highest production of butyric acid was BGM. This can be explained by the fact that the BGM media contains prebiotics like inulin. Similar in vitro studies with inulin have also shown this contributes to the highest average production of butyric acid (Carlson et al. 2017).

The profiles of microbial communities in the four groups indicated different patterns during 48 h culture. The alpha diversity is widely reported as an indicator of intestinal microbial communities state due to its relationship with productivity, functioning and stability (Reese and Dunn 2018). Compared with other three groups, intestinal microbial diversity, as estimated by Shannon index and observed OTUs, was significantly (p < 0.05) higher in BHI group. We observed that a large amount of unique OTUs in the 0 h samples, indicating that those OTUs cannot be cultured during 48 h culture (Fig. 3C–F). We speculate that differences between the in vivo and in vitro environments were the cause of OTUs lost. In vitro batch culture provides a way to culture human intestinal microbiota under simulated physiological conditions, while in vitro batch culture cannot mimic the interactions between microbiota and the host. Moreover, a higher number of OTUs were retained in the BHI group than other groups during the 48 h culture (Fig. 3). These results indicated that BHI media could maintain intestinal microbial diversity during in vitro culture.

At the genus level, *Bacteroides*, *Lachnospira*, *Streptococcus* and *Klebsiella* were the dominant bacteria in the BHI group in LEfse analysis. *Bacteroides* is one of the most abundant genera in the gut and has a positive correlation with propionic acid production (Nakano et al. 2013), and *Lachnospira* also plays an important role in SCFA production (Bang et al. 2018). Previous studies have reported that *Streptococcus* and *Klebsiella* were widely distributed in the human skin, mouth and intestine, and *Streptococcus* can degrade digested proteins into peptides in human gut (Macfarlane et al. 1988; Sekirov et al. 2010; Brisse et al. 2006). The relative abundance of *Bifidobacterium*, *Prevotella*, *Citrobacter* and *Coprococcus* in the GMM group was highest when compared to those in other groups. *Bifidobacterium* resides naturally in the gastrointestinal tract of healthy human adults and comprises a unique genus of bacteria, in which gas is not formed as an end product of metabolism (Routy et al. 2018). *Bifidobacterium* may exert multiple positive effects on human health ranging from prevention of obesity and infections to resolution of ulcerative colitis via the down regulation of TNF-α and IL-1 (Routy et al. 2018). *Prevotella* could improve human glucose metabolism and increase glycogen storage (Kovatcheva-Datchary et al. 2015). *Citrobacter* is the generally opportunistic pathogen and is among the leading causes of morbidity and mortality worldwide (Seo et al. 2017). The previous study found that the patients who suffered irritable bowel syndrome had minute *Coprococcus* (Kassinen et al. 2007). FAB group showed a significantly higher relative abundance of *Clostridium* sensu stricto and *Enterococcus* when compared to those in other three groups. *Clostridium* sensu stricto is usually regarded as the opportunistic pathogen (Zhou et al. 2018). The genus *Enterococcus* is a group of lactic acid bacteria, and they are sometimes associated with infections in humans (Fusco et al. 2017). Furthermore, the higher relative abundance of *Akkermansia* in the BGM group was observed in this study. *Akkermansia* is considered as next-generation probiotics for their anti-inflammatory properties, and is typically closely associated with the protective mucous lining of the human intestine (Schneeberger et al. 2015).

Co-occurrence patterns identified using network analysis can reflect the interactions between microbes (Barberan et al. 2012). The network properties offer the possibility for quick and easy comparisons among complex data sets from different culture media to explore how the culture media influence the composition of microbial communities. Nodes that had high edges were often considered to be potential core nodes, if removed, would lead to large changes in the community (Faust et al. 2012). Thus, the results revealed that *Megamonas*, *Ruminococcus*, *Pseudobutyrivibrio*, and *Ruminococcaceae_un and Oscillibacter* were the key genera in the BHI, GMM, FAB and BGM groups, respectively.

Overall, the work presented here is one of few in vitro studies that compares the effects of culture media on the composition and metabolic activity of the human intestinal microbiota; the results showed the intestinal microbiota displayed different growth in the four media. We envision that these results can be used to facilitate human intestinal microbiota in vitro culture.

## Data Availability

Not applicable.

## Abbreviations

SCFA: short chain fatty acid
FMT: fecal microbiota transplantation
BHI: brain heart infusion
GMM: gut microbiota medium
FAB: fastidious anaerobe broth
BGM: bacterial growth media
CDI: *Clostridium difficile* infection
CPCoA: constrained principal coordinates analysis
QIIME 2: quantitative insights into microbial ecology 2
